# DFT and QSAR studies of PTFE/ZnO/SiO_2_ nanocomposite

**DOI:** 10.1038/s41598-022-19490-0

**Published:** 2023-06-15

**Authors:** Hend A. Ezzat, Maroof A. Hegazy, Rasha Ghoneim, Heba Y. Zahran, Ibrahim S. Yahia, Hanan Elhaes, Ahmed Refaat, Medhat A. Ibrahim

**Affiliations:** 1 Space Lab, Solar and Space Research Department, National Research Institute of Astronomy and Geophysics (NRIAG), Helwan, Cairo 11421 Egypt; 2 Nanoscience Laboratory for Environmental and Bio-Medical Applications (NLEBA), Semiconductor Lab., Metallurgical Lab.1., Physics Department, Faculty of Education, Ain Shams University, Roxy, Cairo, 11757 Egypt; 3 Physics Department, Faculty of Women for Arts, Science and Education, Ain Shams University, Cairo, 11757 Egypt; 4 Molecular Spectroscopy and Modeling Unit, Spectroscopy Department, National Research Centre, 33 El-Bohouth St., Dokki, Giza 12622 Egypt

**Keywords:** Nanoscience and technology, Physics

## Abstract

Polytetrafluoroethylene (PTFE) is one of the most significant fluoropolymers, and one of the most recent initiatives is to increase its performance by using metal oxides (MOs). Consequently, the surface modifications of PTFE with two metal oxides (MOs), SiO_2_ and ZnO, individually and as a mixture of the two MOs, were modeled using density functional theory (DFT). The B3LYPL/LANL2DZ model was used in the studies conducted to follow up the changes in electronic properties. The total dipole moment (TDM) and HOMO/LUMO band gap energy (∆E) of PTFE, which were 0.000 Debye and 8.517 eV respectively, were enhanced to 13.008 Debye and 0.690 eV in the case of PTFE/4ZnO/4SiO_2_. Moreover, with increasing nano filler (PTFE/8ZnO/8SiO_2_), TDM changed to 10.605 Debye and ∆E decreased to 0.273 eV leading to further improvement in the electronic properties. The molecular electrostatic potential (MESP) and quantitative structure activity relationship (QSAR) studies revealed that surface modification of PTFE with ZnO and SiO_2_ increased its electrical and thermal stability. The improved PTFE/ZnO/SiO_2_ composite can, therefore, be used as a self-cleaning layer for astronaut suits based on the findings of relatively high mobility, minimal reactivity to the surrounding environment, and thermal stability.

## Introduction

Polyethylene naphthalate (PEN), polyethylene terephthalate (PET), and polytetrafluoroethylene (PTFE) are among the well-known polymers owing to their corrosion resistance and electrical characteristics, as well as their low coefficient of friction, high temperature resistance, and cost efficiency^1^. Super hydrophobic materials such as fluoropolymers have become an extraordinary advantage in a number of applications, including self-cleaning, anti-icing, anti-corrosion, and protective properties such as high efficiency^2,3^. Smart textiles are also considered a new trend based on fluoropolymers with nanomaterials that might be used to improve textiles such as space suits and gloves, medical applications such as surgical garments, and usage in smart hospitals^4,5^. PTFE is a polymer matrix with a low surface energy and is chemically and thermally stable^6^. Smart materials innovation might be employed in space applications like spacesuits and storage by modifying materials to react to changes in ambient temperature or even body temperature^7–9^. PTFE properties such as anticorrosive properties are becoming increasingly significant, particularly in the aerospace industry. The significance arises from its benefits, which are critical for protecting materials from cracking and/or corrosion in the harsh aerospace environment. Consequently, utilizing an anti-corrosion substance to adequately protect and prevent astronaut suits from rust and corrosion is a novel approach^10–12^. The effective manufacturing of a wide range of sensors is enabled by the use of PTFE as a substrate for the growth of ZnO nanotubes, as well as its mechanical, physical, and chemical characteristics^13^. Nano silica is a type of ceramic material that has several unique properties, including high hardness, corrosion resistance, and outstanding electrical insulation^14^. All of these properties combine to make SiO_2_ and PTFE a unique material that is ideal for a wide range of technical applications^15^. In addition, combining SiO_2_ with semiconductor oxide materials such as ZnO^16^, TiO_2_^17^, Fe_2_O_3_^18^, and CuO^19^ improves the self-cleaning, anti-corrosion, anti-reflective, and magnetic characteristics of nanocomposite materials.

PTFE/SiO_2_ composite has a superhydrophobic surface when compared to PTFE membranes^20^. PTFE/SiO_2_ nanofibers have shown to be a reliable invention for excellent thermal and chemical stability^21^. Doping PTFE with SiO_2_ reduces PTFE porosity deformation while simultaneously increasing the material's tensile strength and endurance. As the quantity of SiO_2_ in the PTFE/SiO_2_ composite increased, so did the mechanical characteristics^22^. The tribological efficiency of PTFE/SiO_2_/Epoxy composites was also studied^23^. The effect of Al_2_O_3_ nanoplatelets on the PTFE matrix was observed to increase thermal conductivity, thermal stability, and enhance the mechanical properties with significantly enhanced electrical properties^24^. Furthermore, the electrical characteristics of the PANI/PTFE/GO^25^ and PTFE/CuO/G^26^ hybrid composites have shown an improvement to be employed in the fabrication of electrochemical instruments. The ZnO/SiO_2_/PTFE film over glass was made with anti-icing properties, corrosion resistance, and insulation properties, thus acting as an anti-icing surface^27^. Furthermore, some derivatives of PTFE, such as Teflon FEP, are used as thermal control layers for the Hubble Space Telescope (HST)^28–30^. Teflon FEP suffers from corrosion due to the space environment in the low Earth orbit (LEO)^31^, thus exposing the components in space to damage and corrosion^32,33^.

Consequently, improving PTFE and its derivatives has been a valuable point of study for space applications^34,35^.

Some physical parameters such as TDM, HOMO/LUMO band gap energy (∆E), and MESP are regarded as efficient predictors of electrical properties as well as the reactivity of the studied interactions^36–40^. Furthermore, QSAR provides important information on molecular behaviors, by evaluating the chemical, biological, and physical activities of molecules used in numerous applications^41–44^. By examining the hydrophobicity of a given structure and its behavior with its surroundings, DFT calculations using various physical parameters are commonly used to confirm superhydrophobic and exceptional performance in anti-corrosion, anti-icing, and self-cleaning^45,46^.

Based on the described features of PTFE and its nanocomposites, the current work is conducted to investigate the physicochemical properties such as anti-corrosion and self-cleaning of surface modified PTFE utilizing ZnO and SiO_2_ individually and combined. DFT calculations were performed using the B3LYP/LANL2DZ model to track changes in TDM, ∆E and mapping MESP, all of which representing changes in electronic characteristics. Electronic property information associated with QSAR descriptors were utilized to evaluate electronic characteristics, as well as thermal, physical, and chemical stability for potential usage as corrosion-inhibiting layers in astronaut suits.

## Computational methods

PTFE model molecule interacted with two nano-MOs, ZnO and SiO_2_, separately and as a mixture were calculated using the GAUSSIAN 09 software (Gaussian, Inc.: Wallingford, CT, USA)^47^ at the Molecular Spectroscopy and Modeling Unit, National Research Centre (NRC), Egypt. DFT:B3LYP/LANL2DZ was used to optimize the proposed models^48–50^. Electronic properties were studied, including TDM, ΔE and MESP. Furthermore, SCIGRESS 3.0 software^51^ was used to study the chemical and thermal stability for all the model structures. QSAR parameters were calculated for model structures using MO-G at PM6 level of theory^52^.

## Results and discussion

### Building PTFE model structures

**Figure 1:**
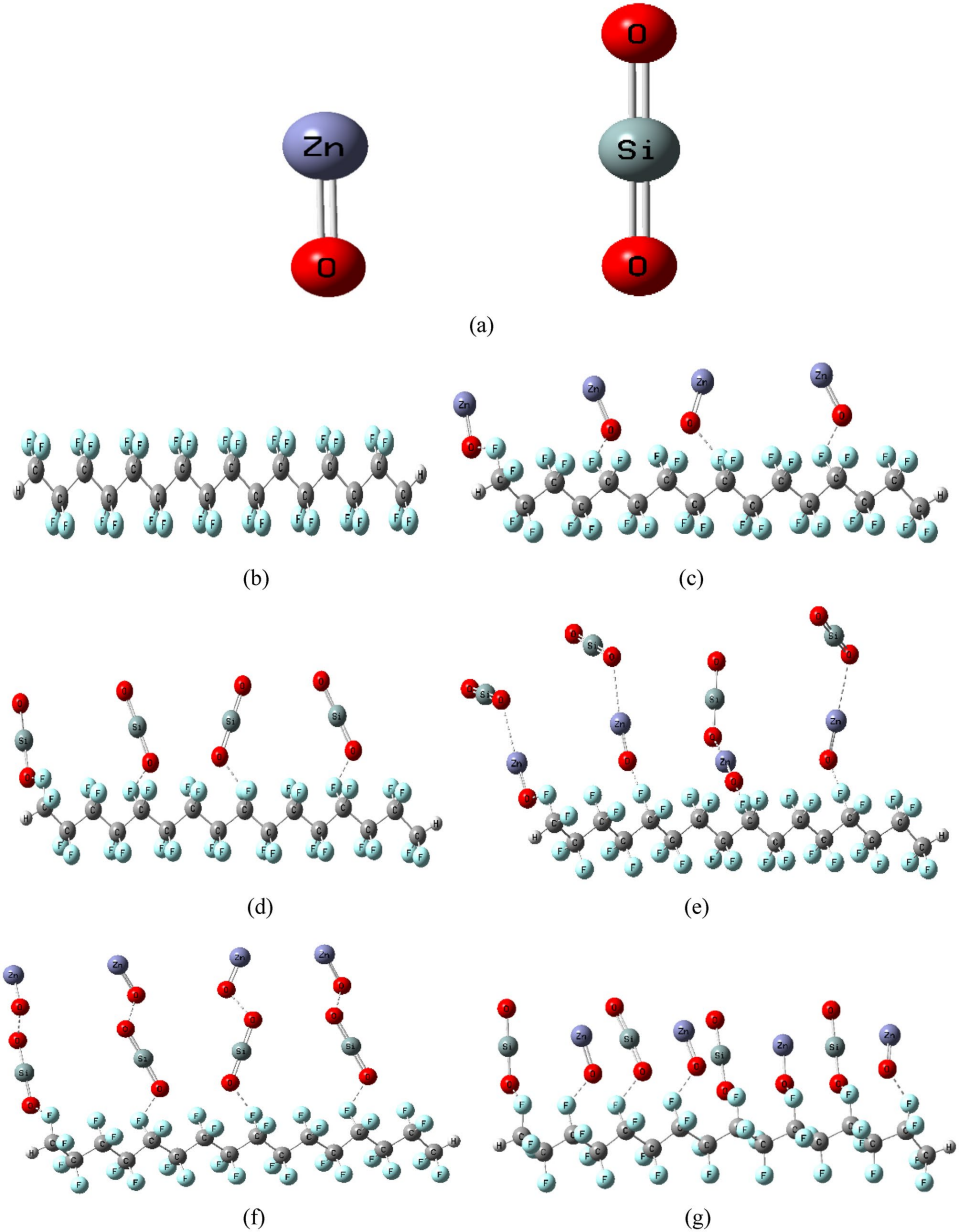
Optimized structure for PTFE and PTFE interaction with 4 ZnO, 4 SiO_2_ and a combination between the two MOs as (**a**) MOs (ZnO and SiO_2_), (**b**) PTFE, (**c**) PTFE/4ZnO, (**d**) PTFE/4SiO_2_, (**e**) PTFE/4ZnO/4SiO_2_, (**f**) PTFE/4SiO_2_/4ZnO and (**g**) PTFE/(4ZnO&4SiO_2_).

**Figure 2:**
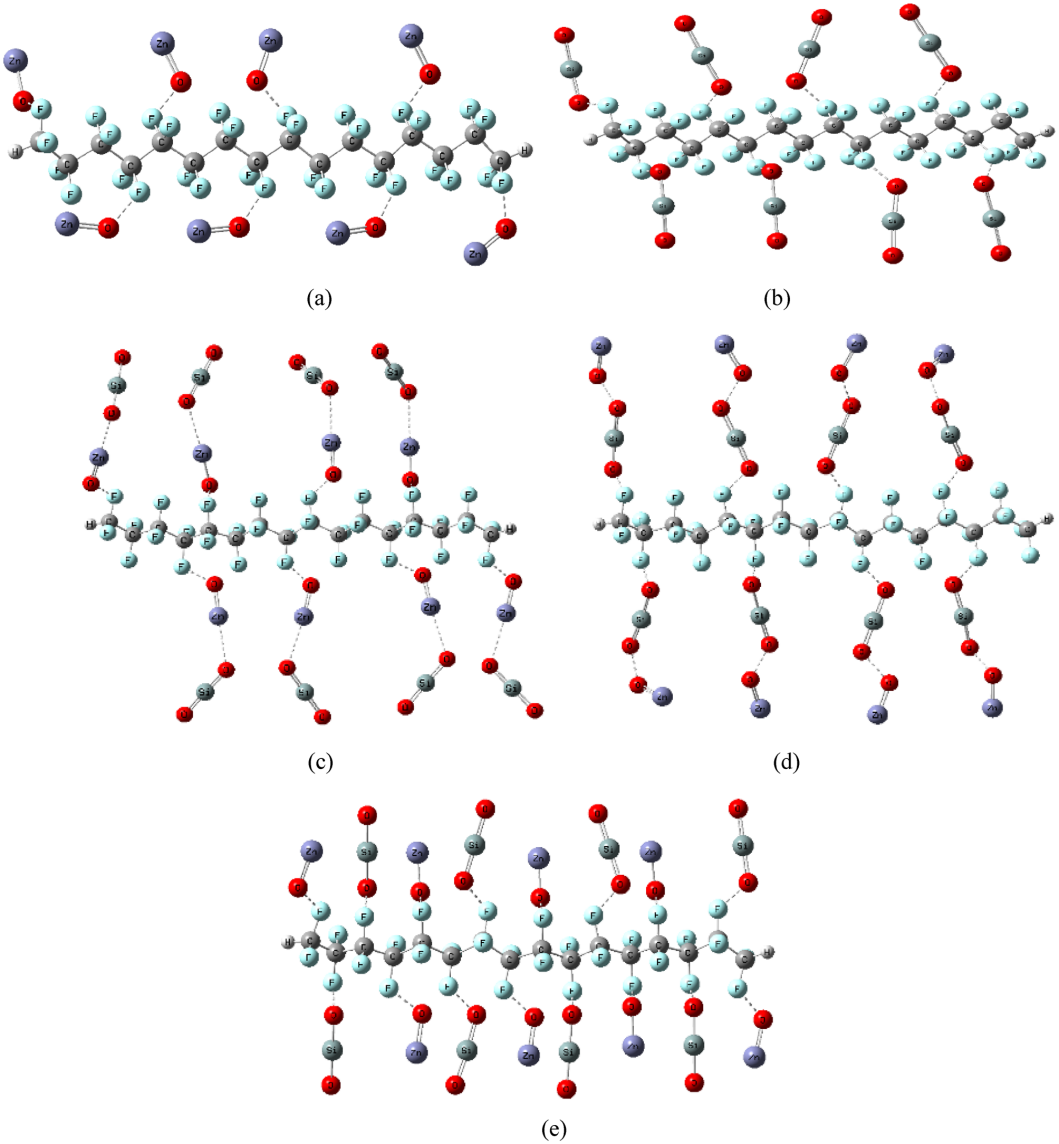
Optimized structure for PTFE and PTFE interaction with 8 ZnO, 8 SiO_2_ and a combination between the two MOs as (**a**) PTFE/8ZnO, (**b**) PTFE/8SiO_2_, (**c**) PTFE/8ZnO/8 SiO_2_, (**d**) PTFE/8 SiO_2_/8ZnO and (**e**) PTFE/(8ZnO&8 SiO_2_).

A model of molecules simulating PTFE coated with MOs to promote hydrophobicity, anticorrosion, and self-cleaning qualities^53^. MOs including ZnO and SiO_2_ have been suggested as coating layers because of their anticorrosion and self-cleaning properties^54,55^. Consequently, the model of the smallest set of PTFE chemical units, representing a PTFE polymer chain, is designed to interact with ZnO and SiO_2_ both individually and combined. The interaction of PTFE with MOs takes place via the oxygen atom of the MO^26,56^. Because PTFE interacts chemically via its active sides, and since it has four equal active sides according to the chemical formula C_2_F_4_; therefore, any fluorine (F-) atom can interact with other chemical structures. As indicated in Fig. 1a,b, the model of the smallest unit representing the two MOs (ZnO and SiO_2_) and the PTFE polymer chain, was made up of four units of C_2_F_4_ that were designed to interact with the suggested two MOs. First, the PTFE chain was designed to interact with four units of ZnO and four units of SiO_2_, separately, coated on one side, as shown in Fig. 1c,d, respectively. After that, the PTFE chain is designed to interact with a combination of four units of ZnO and four units of SiO_2_ covered layer by layer as shown in Fig. 1e. Figure 1f depicts the PTFE chain's model structure as it interacts with a combination of four units of SiO_2_ and four units of ZnO covered layer by layer on its surface. Figure 1g depicts the final model structure for the PTFE chain in interaction with a single mixed layer of four ZnO and four SiO_2_ units. Increasing the quantity of nanoparticles on the polymer's surface has a significant impact on the electrical properties of the polymer's matrix^57^. Following that, the PTFE chain is next expected to be coated from both sides, as previously done with four units of ZnO and four units of SiO_2_ individually and combined, as shown in Fig. 2. The electrical characteristics of PTFE/MOs were then examined for the proposed interaction mechanisms by studying the calculated TDM, ΔE and MESP maps.

### HOMO/LUMO orbital distribution

**Figure 3:**
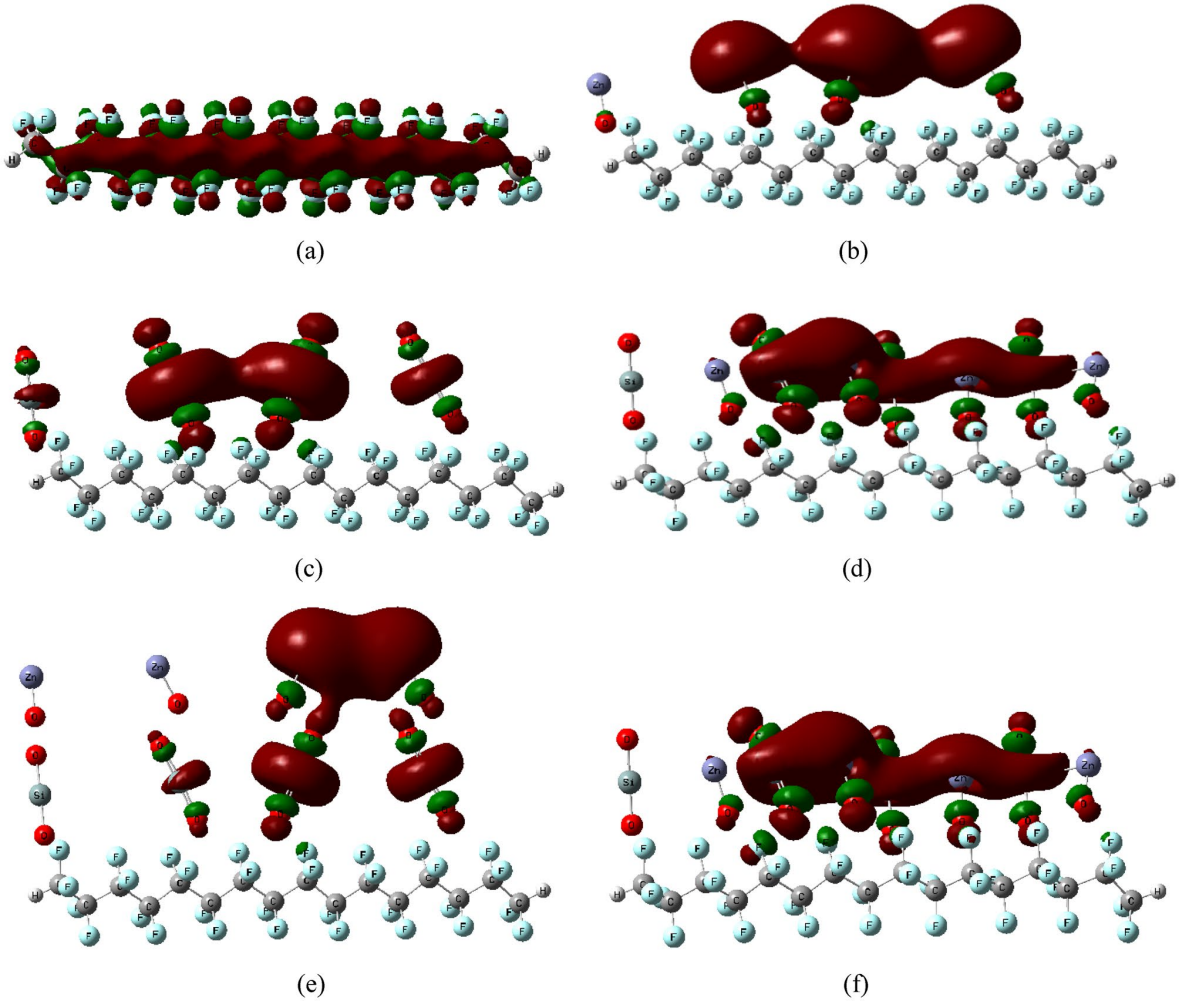
DFT:B3LYP/LANL2DZ calculated HOMO/LUMO orbital distribution of PTFE and PTFE interaction with 4ZnO, 4 SiO_2_ and a combination between the two MOs as (**a**) PTFE, (**b**) PTFE/4ZnO, (**c**) PTFE/4SiO_2_, (**d**) PTFE/4ZnO/4SiO_2_, (**e**) PTFE/4SiO_2_/4ZnO and (**f**) PTFE/(4ZnO&4SiO_2_).

Figure 3 illustrates the HOMO/LUMO orbital distribution of PTFE and its interactions with 4ZnO, 4SiO_2_, and their hybrids. The HOMO/LUMO orbital distribution of the four PTFE chains is demonstrated in Fig. 3a to be spread across the chain. When PTFE interacted with ZnO and SiO_2_ on one side, the HOMO/LUMO orbitals were rearranged as in Fig. 3b–f for all interaction cases and localized around the MO atoms. HOMO/LUMO orbital distribution reflects the effect of MO on orbital distribution, which in turn reflects on band gap energy changes. TDM and ΔE were also determined for the different forms of interactions. TDM improved from 0.000 for pure PTFE to 16.235, 1.849, 13.008, 17.432 and 11.583 Debye for PTFE/4ZnO, PTFE/4SiO_2_, PTFE/4ZnO/4SiO_2_, PTFE/4SiO_2_/4ZnO and PTFE/(4ZnO&4SiO_2_), respectively, as listed in Table 1. Also, the calculated ΔE was reduced from 8.517 eV for pure PTFE to 1.535, 4.302, 0.690, 1.345, and 0.958 eV for PTFE/4ZnO, PTFE/4SiO_2_, PTFE/4ZnO/4SiO_2_, PTFE/4SiO_2_/4ZnO, and PTFE/(4ZnO&4SiO_2_), respectively. The lowest ΔE was recorded for the PTFE/4ZnO/4SiO_2_ structure, which is an indication for the most probable and stable structure of interaction that could occur for PTFE with the proposed MOs. It is well known that the increase in the reactivity of chemical systems is correlated with their higher calculated TDM and lower calculated ∆E^58^.

**Table 1:** TDM as Debye and band gap energy (ΔE) as eV for PTFE and PTFE interacted with 4ZnO, 4 SiO_2_ and a combination between the two MOs using DFT:B3LYP/LANL2DZ.

Structure	TDM (Debye)	∆E (eV)
PTFE	00.000	8.517
ZnO	05.485	2.602
SiO_2_	00.000	5.094
PTFE/4ZnO	16.235	1.535
PTFE/4SiO_2_	01.849	4.302
PTFE/4ZnO/4SiO_2_	13.008	0.690
PTFE/4SiO_2_/4ZnO	17.432	1.345
PTFE/(4ZnO&4SiO_2_)	11.583	0.958

**Figure 4:**
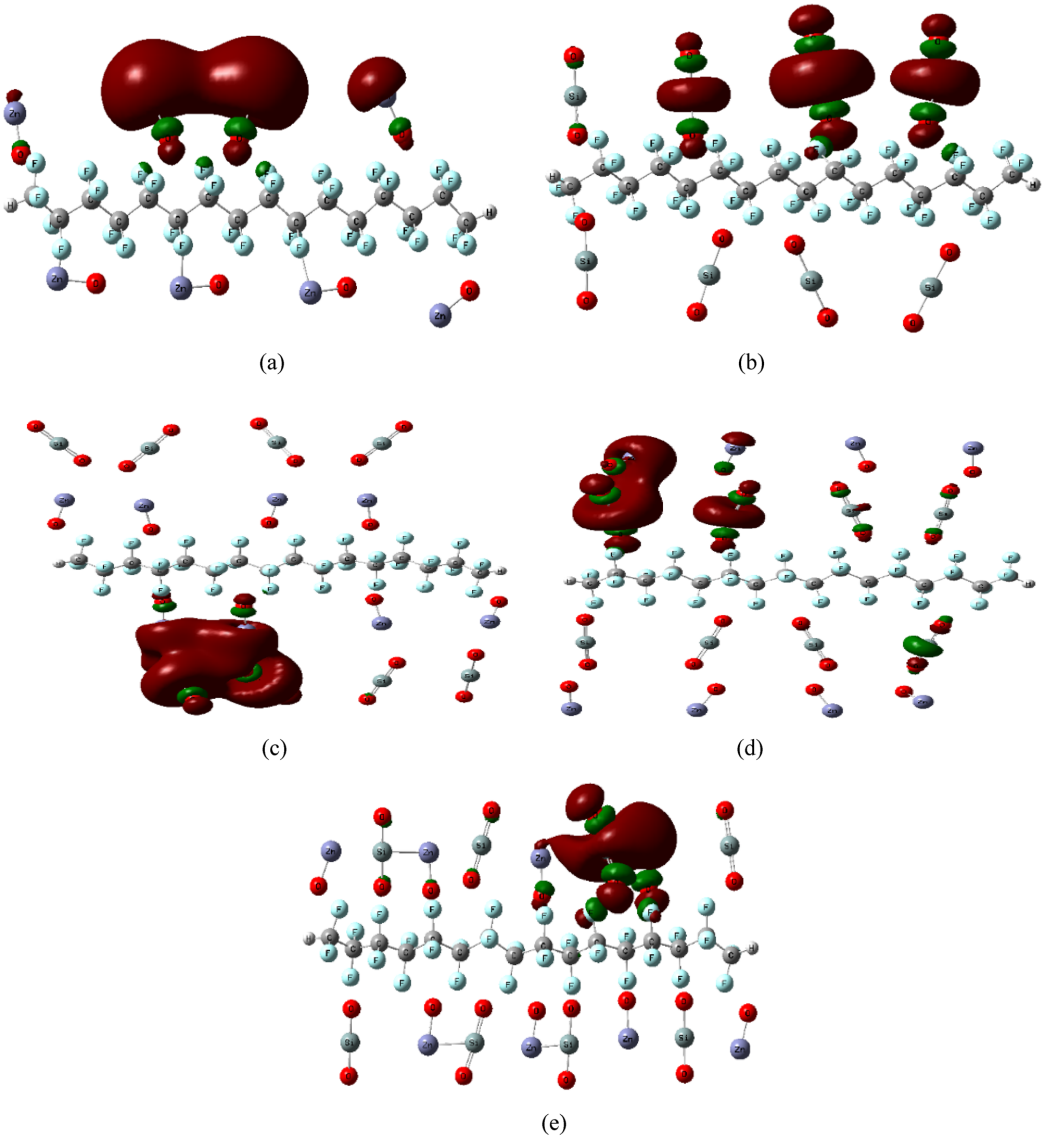
DFT:B3LYP/LANL2DZ calculated HOMO/LUMO orbital distribution of PTFE interaction with 8ZnO, 8SiO_2_ and a combination between the two MOs as (**a**) PTFE/8ZnO, (**b**) PTFE/8SiO_2_, (**c**) PTFE/8ZnO/8SiO_2_, (**d**) PTFE/8SiO_2_/8ZnO and (**e**) PTFE/(8ZnO&8 SiO_2_).

Figure 4 shows the calculated HOMO/LUMO orbital distribution of PTFE interaction with 8 ZnO, 8 SiO_2_ and a combination of the two MOs. In these cases of interactions, as shown by HOMO/LUMO orbitals rearranged and localized around the MO on one side only, at the top and/or down the PTFE chain (at the top in Fig. 4a,b,d,e but down in the 4c case).The TDM and ΔE were determined for all the studied structures and are listed in Table 2. As shown in the table, TDM improved from 0.000 corresponding to pure PTFE to 32.934, 0.867, 7.844, 10.605 and 6.963 Debye for PTFE/8ZnO, PTFE/8SiO_2_, PTFE/8ZnO/8SiO_2_, PTFE/8SiO_2_/8ZnO, and PTFE/(8ZnO&8SiO_2_), respectively. While ΔE was observed to decrease from 8.517 eV for pure PTFE to 0.163, 3.253, 0.273, 0.860, and 0.368 eV for PTFE/8ZnO, PTFE/8SiO_2_, PTFE/8ZnO/8SiO_2_, PTFE/8SiO_2_/8ZnO, and PTFE/(8ZnO&8SiO_2_), respectively. The highest value of TDM and the lowest value of ΔE were reported for PTFE/8ZnO and PTFE/8ZnO/8SiO_2_ as an indication of the interactions that enhanced the electrical characteristics of the PTFE the most. From all the results, the most enhanced PTFE structure interacted with 4 unit's MOs was that of PTFE/4ZnO/4SiO_2_. By increasing nanoparticles to 8 units, the most enhanced structure was that for PTFE/8ZnO and PTFE/8ZnO/8SiO_2_. This means that the electronic behavior of the PTFE polymer is modified with increasing the number of units of MOs.

**Table 2:** TDM as Debye and band gap energy (ΔE) as eV of PTFE and PTFE interaction with 8 ZnO, 8 SiO_2_ and a combination between the two MOs using DFT:B3LYP/LANL2DZ level of theory.

Structure	TDM (Debye)	∆E (eV)
PTFE	00.000	8.517
ZnO	05.485	2.602
SiO_2_	00.000	5.094
PTFE/8ZnO	32.934	0.163
PTFE/8SiO_2_	00.867	3.253
PTFE/8ZnO/8SiO_2_	10.605	0.273
PTFE/8SiO_2_/8ZnO	07.844	0.860
PTFE/(8ZnO&8SiO_2_)	06.963	0.368

Generally, as TDM and ΔE for the interaction of PTFE with MOs are enhanced by increasing the number of MOs units, one can say that as the quantity of nanoparticles increases, the electrical properties of the suggested model structure improve.

### Molecular electrostatic potential (MESP)

**Figure 5:**
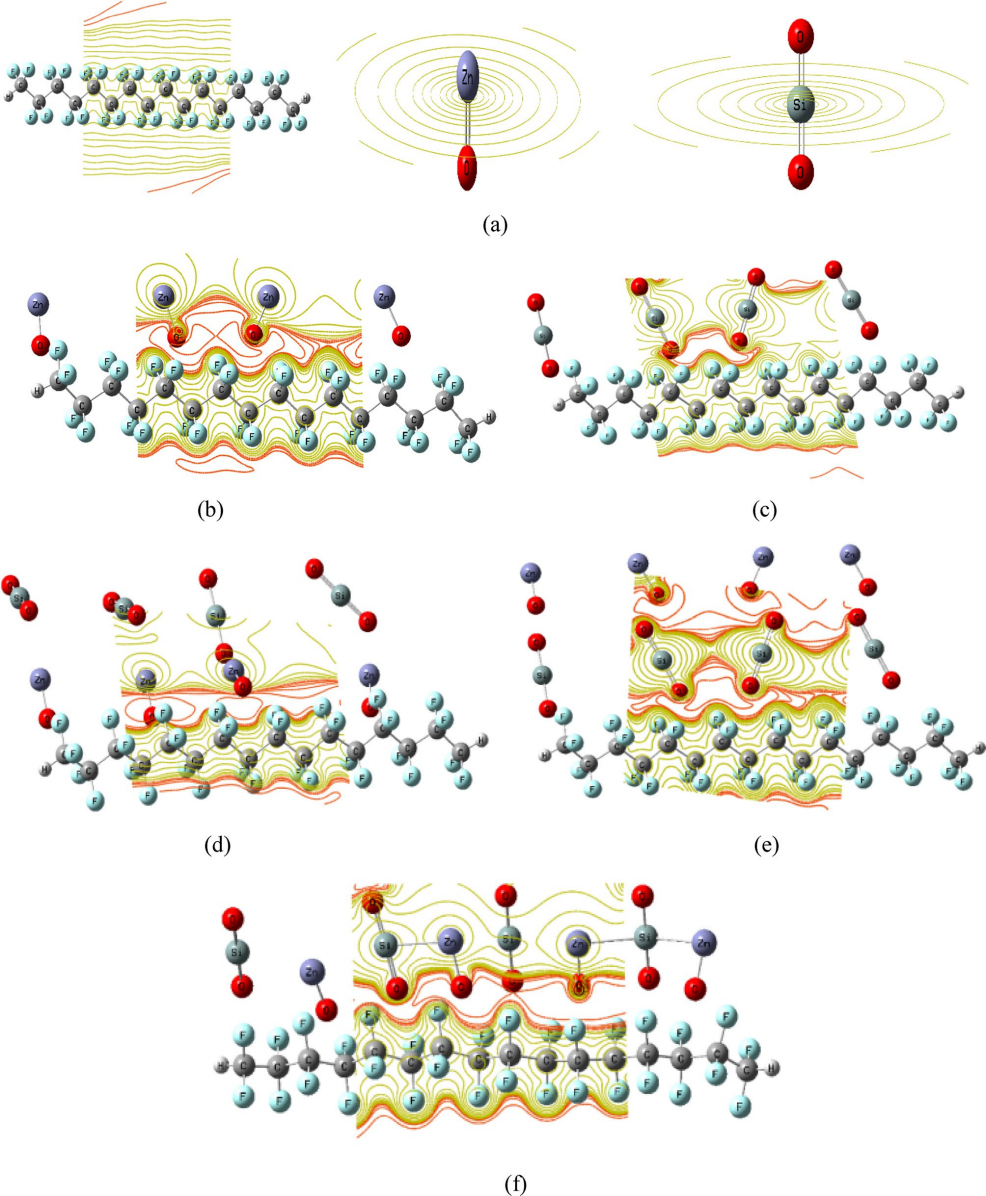

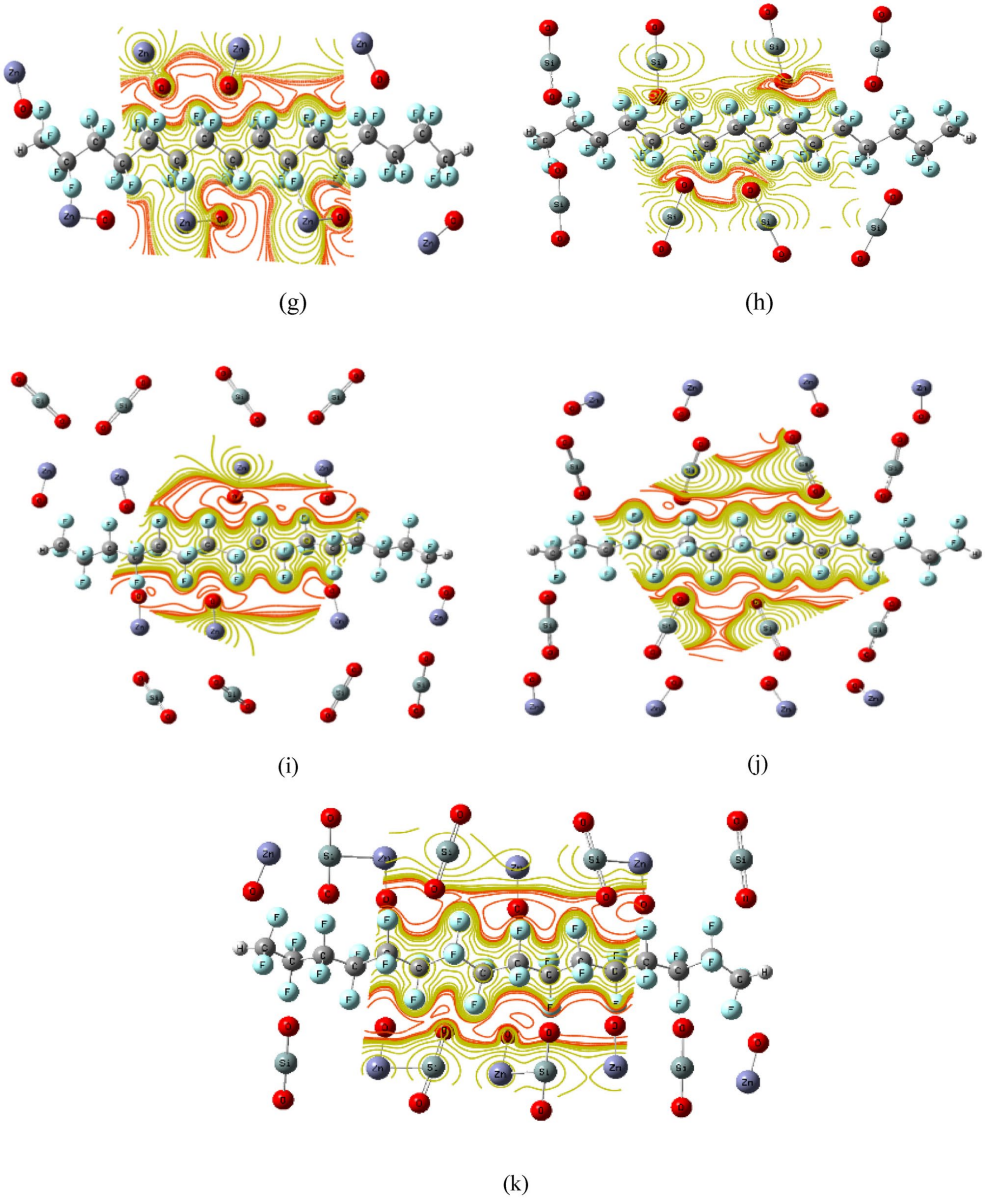
DFT:B3LYP/LANL2DZ calculated MESP for PTFE and PTFE interaction with ZnO, SiO2 and a combination between the two MOs as (**a**) PTFE, ZnO and SiO_2_, (**b**) PTFE/4ZnO, (**c**) PTFE/4SiO_2_, (**d**) PTFE/4ZnO/4SiO_2_, (**e**) PTFE/4SiO_2_/4ZnO, (**f**) PTFE/(4ZnO&4 SiO_2_), (**g**) PTFE/8ZnO, (**h**) PTFE/8SiO_2_, (**i**) PTFE/8ZnO/8 SiO_2_, (**j**) PTFE/8 SiO_2_/8ZnO and (**k**) PTFE/(8ZnO&8 SiO_2_).

The MESP map displays the distributions of the nearby charges, nucleus, and density of electrons at a particular position, represented with color variation as Red > Orange > Yellow > Green > Blue. The colour difference represented as red on the MESP surface refers to the richest charge area, the colour difference represented as blue refers to the poorest charge region, and the colour difference described as green represents zero electrostatic potential. The strongest potential is commonly found in red regions, whereas the weakest potential is found in blue regions^59^. MESP mapping was calculated for all studied structures at the same level of theory. Figure 5 shows the MESP for PTFE/4ZnO, PTFE/4SiO_2_, PTFE/4ZnO/4SiO_2_, PTFE/4SiO_2_/4ZnO, PTFE/(4ZnO&4SiO_2_), PTFE/8ZnO, PTFE/8SiO_2_, PTFE/8ZnO/8SiO_2_, PTFE/8SiO_2_/8ZnO and PTFE/(8ZnO&8SiO_2_), which displayed a map for the interaction status of nucleophilicity.

Figure 5a shows the MESP map for all considered PTFE, ZnO and SiO_2_ interactions colored with intermediate colors between orange and yellow, with a plane for the PTFE chain and perpendicular in the case of MOs, which represents less electrostatic repulsion. The MESP results revealed that these structures were exceptionally stable, with the MESP surface appearing in yellow, and there was still no chance of interfering with others, and representing more chemical equilibrium. Figure 5b–k demonstrate the interaction of PTFE with MOs. The red colour spread on the up and down terminals of the polymer, indicating that PTFE's reactivity increased, and MOs enhanced PTFE's active sides. When PTFE interacted with 4SiO_2_, 4ZnO/4SiO_2_, 4SiO_2_/4ZnO, (4ZnO&4SiO_2_), 8SiO_2_, 8ZnO/8SiO_2_, 8SiO_2_/8ZnO, and (8ZnO&8SiO_2_), low-potential red regions were localized mainly around the oxygen atom of MO. Whereas when PTFE interacted with 4ZnO and 8ZnO, the red regions were spread across the polymer and increased on the other side of it. These results of MESP are in good agreement with the results of TDM and ΔE. As a result, PTFE's electrical properties improved, and it may now be employed in a variety of fields of applications, such as a corrosion-inhibiting layer for astronaut suits.

### Quantitative structure activity relationship (QSAR)

Table 3 defines QSAR descriptors trying to describe PTFE relationships with MOs as PTFE/4ZnO, PTFE/4SiO_2_, PTFE/4ZnO/4SiO_2_, PTFE/4SiO_2_/4ZnO, PTFE/(4ZnO&4SiO_2_), PTFE/8ZnO, PTFE/8SiO_2_, PTFE/8ZnO/8SiO_2_, PTFE/8SiO_2_/8ZnO and PTFE/(8ZnO&8SiO_2_). Descriptors are summarized as total energy (TE) as Kcal/mol, heat formation (HF) as Kcal/mol, ionization potential (IP) as eV, log P, polarizability as A^3^, molar refractive (MR) and molecular weight (MR) as au. Firstly, TE is stated to describe the stability of the system, and that reducing TE values takes the structure toward stability^60^. TE for PTFE was − 17,005.449 kcal/mol. In case of PTFE interacted with 4 units of MOs, TE changed for PTFE/4ZnO, PTFE/4SiO_2_, PTFE/4ZnO/4SiO_2_, PTFE/4SiO_2_/4ZnO and PTFE/(4ZnO&4SiO_2_) to − 18,409.137, − 20,012.627, − 10,891.274, − 931.899 and − 932.368 kcal/mol respectively. While the TE for PTFE/8ZnO, PTFE/8SiO_2_, PTFE/8ZnO/8SiO_2_, PTFE/8SiO_2_/8ZnO and PTFE/(8ZnO&8SiO_2_) were changed to − 11,544.339, − 23,034.521, − 25,976.165, − 2414.112, and − 25,973.368 kcal/mol respectively. From the calculated TE, PTFE/8ZnO/8SiO_2_ found to be the most stable and most probable structure. As well, HF is a significant thermal descriptor that defines the energy produced in the form of heat, as the atoms that exist at potentially infinite distances are linked and form a molecule^61^, even though HF may be clarified through the difference observed in the enthalpy during the formation of a single mole of a substance from its components. This occurs in its natural and full balance under the atmospheric characteristics of a particular temperature. For PTFE, HF was equal to − 1570.772 kcal/mol. In the case of PTFE interaction with MOs, the calculated HF for PTFE/4ZnO, PTFE/4SiO_2_, PTFE/4ZnO/4SiO_2_, PTFE/4SiO_2_/4ZnO, and PTFE/(4ZnO&4SiO_2_) were changed to − 1826.101, − 1963.831, − 2457.839, − 2598.762, and − 2266.677 kcal/mol. While in the case of PTFE/8ZnO, PTFE/8SiO_2_, PTFE/8ZnO/8SiO_2_, PTFE/8SiO_2_/8ZnO and PTFE/(8ZnO&8SiO_2_), HF changed to be equal to − 1871.705, − 2597.869, − 3128.116, − 2372.734, and − 3300.601 kcal/mol, respectively. Accordingly, the most probable structure to be formed, which needed a low energy value for formation, was PTFE/8ZnO/8SiO_2_ and PTFE/(8ZnO&8SiO_2_).

**Table 3:** QSAR calculations for PTFE and PTFE interaction with of ZnO, SiO_2_ and the combination of both MOs at using descriptors as Total Energy (TE) as Kcal/mol, Heat of formation (HF) as Kcal/mol, Ionization potential (IP) as eV, Log P, Polarizability as A^3^, Molar refractive (MR), molecular weight (MW) as au, which were calculated at PM6 semiempirical method.

Sample	TE (Kcal/mol)	HF (Kcal/mol)	IP (eV)	Log P	Polarizability (A^3^)	MR	MW (au)
PTFE	− 17,005.449	− 1570.772	− 12.980	12.374	28.228	76.442	802.136
PTFE/4ZnO	− 18,409.137	− 1826.101	− 9.417	10.968	44.629	1127.770	1127.770
PTFE/4SiO_2_	− 20,012.627	− 1963.531	− 9.446	9.562	38.934	1042.473	1042.473
PTFE/4ZnO/4SiO_2_	− 931.899	− 2457.839	− 8.953	8.157	49.835	1368.107	1368.107
PTFE/4SiO_2_/4ZnO	− 10,891.274	− 2598.762	− 8.746	8.157	48.952	1368.107	1368.107
PTFE/4ZnO&4SiO_2_	− 932.368	− 2266.677	− 10.573	8.157	53.616	1368.107	1368.107
PTFE/8ZnO	− 11,544.339	− 1871.705	− 9.737	9.562	38.636	1453.403	1453.403
PTFE/8SiO_2_	− 23,034.521	− 2597.869	− 11.648	6.751	49.139	1282.810	1282.810
PTFE/8ZnO/8SiO_2_	− 25,976.165	− 3128.116	− 8.733	3.940	76.588	1934.078	1934.078
PTFE/8SiO_2_/8ZnO	− 2414.112	− 2372.734	− 8.420	3.940	77.496	1934.078	1934.078
PTFE/8ZnO&8SiO_2_	− 25,973.368	− 3300.601	− 10.392	3.940	79.960	1934.078	1934.078

Another important descriptor is IP, which is defined as the energy required for the material to be ionized. The IP described by Dewar and Morita using the following equation: IP = a + bq + cq, that a, b is the variational parameter defined as a^2^ + b^2^ = 1, charge of an atom in a molecule (q), and electron density of an atom in a molecule (C)^62,63^. IP was calculated for all PTFE models of interaction. The importance of IP value is that it reflects the reactivity of the studied structures. The IP value is inversely proportional to the compound's reactivity, which means that the reactivity of a certain chemical compound increases as the IP value decreases^64^. The IP value recorded for PTFE was equal to − 12.980 eV. When PTFE interacted with 4 MOs, the IP changed to − 9.417, − 9.446, − 8.953, − 8.746 and − 10.573 eV for PTFE/4ZnO, PTFE/4SiO_2_, PTFE/4ZnO/4SiO_2_, PTFE/4SiO_2_/4ZnO and PTFE/(4ZnO&4SiO_2_), respectively. In the case of PTFE interacted with 8 MOs, IP changed to − 9.737, − 11.648, − 8.733, − 8.420 and − 10.392 eV for PTFE/8ZnO, PTFE/8SiO_2_, PTFE/8ZnO/8SiO_2_, PTFE/8SiO_2_/8ZnO, and PTFE/(8ZnO&8SiO_2_), respectively. According to the obtained data, there was no significant change in IP value. The PTFE/8ZnO/8SiO_2_ structure had the lowest reactive structure with the surrounding environment and the most thermally enhanced structure. The chemical structure was described by the logarithm of the partition coefficient (log P). Accordingly, the log p value for a compound is the logarithm (base 10) of the partition coefficient (p), which is defined as the ratio of the compounds' organic to aqueous phase concentration as in the equation^65^

$$\mathrm{P}= \frac{Concentration\,\, in \,\,Organic}{ Concentration \,\,in \,\,Aqueous}.$$

It calculates the solubility of the substance even in an organic solution or in aqueous solvents. Positive log P values define the hydrophobic structures while negative values indicate hydrophilic structures^66^. All proposed models recorded a positive log P, which is an indication that the structures are hydrophobic and have not affected the surrounding environment. The lowest value of log P reflects a more polar and increase in hydrophobicity of the compound, which confirms the ability to act as a self-cleaning material. Self-cleaning surfaces have sparked significant interest in industrial applications, particularly in the aerospace industry. Self-cleaning super hydrophobic coatings, such as silicones, fluorocarbons, organic materials, and inorganic materials, are sensitive to the accumulation of ice, water, and other contaminants, as well as having a hard, wear-resistant, and phobic coating for an aerodynamic surface to improve de-icing properties via low-pressure plasma vapour deposition technologies. So, enhancing the hydrophobicity of the studying material makes it a promising material to be used as a self-cleaning surface, which is an important application in the aerospace field^67,68^. The lowest values of log P were recorded for PTFE/8ZnO/8SiO_2_, PTFE/8SiO_2_/8ZnO and PTFE/(8ZnO&8SiO_2_). Consequently, polarizability is a basic property that determines how the chemical formula can be polarized in response to varying forces. Representing the responsiveness of the structural factors affecting the volume, the molar refractor is a descriptor which can specify the overall polarization of the mole^69^. The greater the molar refractor, the greater the stability of the structures, which were recorded for PTFE/8ZnO/8SiO_2_, PTFE/8SiO_2_/8ZnO, and PTFE/(8ZnO&8SiO_2_).

In summary, the obtained results regarding the most expected interaction between PTFE and MOs, suggested a coating of ZnO with SiO_2_ on PTFE, particularly PTFE/8ZnO/8SiO_2_ layer by layer. PTFE/8ZnO/8SiO_2_ is the most probable way of interaction based on its physical, chemical, and thermal stability. These enhancements serve as a corrosion-inhibiting and self-cleaning layer for astronaut suits owing to its lower response with the surroundings and its higher polarity and hydrophobic nature.

## Conclusion

DFT calculations of PTFE chains modified with 4 and 8 units of nano-MOs including ZnO, SiO_2_ individually and as a hybrid were subjected to enhanced chemical, physical, and thermal stability. The B3LYPL/LANL2DZ was used to evaluate TDM, ΔE and MESP for PTFE polymer and its interaction with suggested MOs model structures. For the studied structures, QSAR descriptors were calculated using MO-G at the PM6 level of theory to investigate electronic properties as well as thermal, physical, and chemical stability. The TDM and ΔE results for the interaction of PTFE with MOs indicated that the electronic properties of PTFE were improved by increasing the number of MOs units. Furthermore, introducing the two MOs layer by layer improves and keeps the PTFE polymer chain stable. Electronic property calculations showed that the most enhanced PTFE structure, as interacted with 4 units of MOs, was that for PTFE/4ZnO/4SiO_2_. For the interaction of PTFE with 8 units of MOs, PTFE/8ZnO/8SiO_2_ layer by layer presented the best electronic properties results. The MESP maps also confirmed that the studied structure PTFE/8ZnO/8SiO_2_ shows enhancement and redistribution of the charge on the polymer surface. The results of MESP are in good agreement with the results of TDM and ΔE. Furthermore, QSAR data indicated that coating PTFE as PTFE/8ZnO/8SiO_2_ layer by layer improved electronic and thermal stability, and hydrophobicity properties. Correlating the results, it can be concluded that the modified PTFE with ZnO and SiO_2_ layer by layer has innovative features such as thermal, chemical and physical stability with little sensitivity to surrounding materials, which might be employed as an anti-corrosion and self-cleaning layer for astronaut suits.

## Data Availability

The datasets used and/or analyzed during the current study available from the corresponding author on reasonable request.
